# Voluntary Assisted Dying in the Northern Territory Revisited: Reflections on the Legal and Constitutional Affairs Committee's Report

**DOI:** 10.1007/s11673-026-10561-9

**Published:** 2026-03-18

**Authors:** M. E. Okninski, M. Blake

**Affiliations:** 1 Centre for Health Law and Policy, UWA Law School, University of Western Australia, Perth, Australia; 2 Adelaide Law School, College of Business and Law, Adelaide University, Adelaide, Australia; 3 University of Western Australia Law School, Perth, Australia

**Keywords:** Voluntary assisted dying, End-of-life law, Northern Territory, Australia

## Introduction

On September 30, 2025, the Northern Territory’s Senate Standing Committee on Legal and Constitutional Affairs (“the LCAC”) tabled its final report on Voluntary Assisted Dying in the Northern Territory (“LCAC Report”) (Legal and Constitutional Affairs Committee, Legislative Assembly of the Northern Territory 2025), recommending that voluntary assisted dying (“VAD”) legislation be introduced in the NT (xvii). Currently, the Northern Territory is the only Australian jurisdiction without VAD legislation. Given that the Northern Territory was the first jurisdiction both nationally and globally to enact assisted dying legislation (*Rights of the Terminally Ill Act 1995* [NT]), the position has a whiff of irony about it. That legislation was in force for only a year before Commonwealth legislation was introduced prohibiting the Territories from enacting assisted dying laws,^1^ a prohibition which has only recently been revoked (see *Euthanasia Laws 1998* (Cth); *Restoring Territory Rights Act 2022* (Cth); see also Plattner 1997). The recommendation paves the way for the introduction of draft VAD legislation in the NT, some thirty years after the enactment of the *Rights of the Terminally Act 1995* (NT).

In this short paper, we consider key aspects of the LCAC’s Report, focusing on the recommended eligibility requirements for accessing VAD in the Northern Territory, given the fundamental gatekeeper function of these requirements in accessing any proposed scheme. We reference eligibility criteria in other Australian VAD legislative frameworks and conclude that the LCAC’s proposed model for VAD in the Northern Territory, while largely consistent with approaches adopted in other Australian jurisdictions, differs in key areas adopting a more accessible approach to VAD. We argue that this progressive approach is welcome and should be incorporated in the draft VAD legislation.^2^

## Background Context

The *Restoring Territory Rights Act 2022* (Cth) (“*RTRA*”) commenced operation on the December 13, 2022 (s 2), enabling the territories to legislate for assisted dying, therefore establishing legislative parity on this issue with the Australian States. The importance of the Territories being on an equal footing with the States on the matter of assisted dying was a feature of the Federal parliamentary debates (see, e.g., Commonwealth of Australia, Parliamentary Debates, 1 August 2022, 261, 309–311, 313, 317.^3^

Following the passing of the *RTRA*, the Chief Minister of the Northern Territory established an Independent Expert Advisory Panel (“the Panel”) in August 2023 to conduct an inquiry into the legalization of VAD and produce a report which included making recommendations to inform draft legislation. The Report into Voluntary Assisted Dying in the Northern Territory 2024 (“VAD Report 2024”) was tabled in August 2024 proposing 22 recommendations, including that the Northern Territory introduce VAD legislation largely consistent with the frameworks operational in other Australian jurisdictions (26).

The Panel recommended that VAD should only be available for persons aged eighteen years and older with decision-making capacity and diagnosed with a “serious and incurable condition causing intolerable and enduring suffering that cannot be relieved in a manner they feel is acceptable” that is expected to cause death within twelve months (Voluntary Assisted Dying Independent Expert Advisory Panel 2024, 57–59). Other key recommendations included mandatory training for VAD practitioners, the right to raise a conscientious objection, and prohibiting advance care directives for VAD (Voluntary Assisted Dying Independent Expert Advisory Panel 2024, 38–40, 59–60). In May 2025, the LCAC was convened to undertake an inquiry into the Panel’s recommendations.

The LCAC’s inquiry built upon the VAD Report 2024, consulting extensively with the NT community and key stakeholders. Given that more than 30 per cent of the NT population identify as Aboriginal or Torres Strait Islander, ensuring that the diverse First Nation Communities across the Northern Territory were appropriately represented was central to the consultation process (Legal and Constitutional Affairs Committee 2025, 21–22). The LCAC noted that to facilitate engagement in discussion of end-of-life issues, it was important to put measures in place so conversations could be conducted in a culturally appropriate manner, with local interpreters available for face-to-face consultations (Legal and Constitutional Affairs Committee 2025, 13–15).

The LCAC tabled its Final Report on 30 September 2025, putting forward eighty-six recommendations for the Government to consider, including the introduction of VAD legislation in the Northern Territory (xvii, 1).

## Eligibility Criteria Recommendations

Consistent with VAD legislation in every Australian jurisdiction, the LCAC Report recommended that VAD should only be available to adults aged eighteen years and over, who are, throughout the entire process, acting voluntarily and without coercion and who retain decision-making capacity in relation to the decision to access VAD (84, 94, 101) (for discussion on VAD eligibility criteria in the Australian States, see Waller, et al. 2023). In relation to the latter requirement, the LCAC were not supportive of permitting access to VAD pursuant to a request in an advance care directive, although it was noted that this was an evolving area which may need to be reviewed post the introduction of legalization (Legal and Constitutional Affairs Committee 2025, 100–101). Additionally, the committee recommended that requests for VAD not be available on the sole ground of a person experiencing suffering associated with mental illness and/or disability alone, although again this was noted to be an evolving issue (Legal and Constitutional Affairs Committee 2025, 101–103). These recommendations are aligned with the approach taken by all other Australian VAD frameworks.

In one significant aspect, however, the LCAC’s recommendations depart from these other frameworks. The LCAC Report noted that a more flexible approach to decision-making for VAD was essential in the Northern Territory due to the significant number of Aboriginal and Torres Strait Islander communities and the collective decision-making norms and kinship networks at the heart of these (81). It considered that the approach to decision-making in other Australian Jurisdictions, requiring that the person to act voluntary and without coercion, would be problematic for Aboriginal and Torres Strait Islander communities. A more culturally appropriate approach was recommended giving people within these communities the option to “voluntarily request family members and other culturally important decision-makers to be involved in making a VAD decision in accordance with culturally accepted practices of decision-making” (Legal and Constitutional Affairs Committee 2025, 84). In this respect the LCAC Report appears to acknowledge the importance of relational autonomy, an interpretation of the ethical principle of personal autonomy favoured by feminist scholars (see Stoljar and Mackenzie 2022).

### Eligibility Criteria—Underlying Medical Condition

The LCAC further recommended that eligibility should be restricted to individuals diagnosed with a condition that is “advanced, progressive and expected to cause death” which is “causing intolerable and enduring suffering that cannot be relieved in a manner the person feels is acceptable” (Legal and Constitutional Affairs Committee 2025, 88). However, it recommended the adoption of a broader definition of suffering included in other Australian VAD frameworks,^4^ noting that suffering should be defined to mean “mental or physical suffering” including “anticipation or expectation … of future treatment or the progression of the medical condition” (Legal and Constitutional Affairs Committee 2025, 88). Significantly, the LCAC recommended that the definition of the underlying medical condition exclude a life expectancy requirement. Furthermore, it was noted that medical practitioners find it difficult to reliably determine a person’s life expectancy, and the recommended requirement that the underlying medical condition be “advanced, progressive and expected to cause death” was sufficient to prevent any expansion of the law to non-terminal conditions (Legal and Constitutional Affairs Committee 2025, 89), adopting the position taken in the Australian Capital Territory under the recently enacted *Voluntary Assisted Dying Act 2024* (ACT) [s 11(1)(b)].

The exclusion of a life expectancy requirement in the Northern Territory is a significant point of departure from most VAD frameworks enacted throughout Australia, with the exception, as noted above, being the Australian Capital Territory. For example, in Victoria, Western Australia (WA), Tasmania,^5^ South Australia (SA), and New South Wales (NSW), VAD is only available for persons with a life expectancy of twelve months or less if they suffer from a neurodegenerative condition or six months or less for all other conditions (*Voluntary Assisted Dying Act 2017* (Vic) ss 9(1)(d)(iii), 9(4); *Voluntary Assisted Dying Act 2019* (WA) ss 16(1)(c)(ii); *End of Life Choices (Voluntary Assisted Dying) Act 2021* (Tas) s 6(1)(c)(i), (ii); *Voluntary Assisted Dying Act 2021* (SA) ss 26(1)(d)(iii), 26(4); *Voluntary Assisted Dying Act 2022* (NSW) s 16(1)(d)(ii)). While Queensland has a life expectancy requirement, it is twelve months or less for all conditions (*Voluntary Assisted Dying Act 2021* (Qld) s 10(1)(a)(ii)). The recommended absence of a life expectancy requirement indicates a preference for a more accessible VAD framework, one that is also reflected in the LCAC’s views on the imposition of a local residency requirement.

### Eligibility Criteria—Citizenship and Residency Requirements

In terms of citizenship and residency requirements, it was recommended that VAD should be available to Australian citizens or persons who have resided in Australia for at least two continuous years, while also requiring persons to ordinarily reside in the Northern Territory (Legal and Constitutional Affairs Committee 2025, 93–94). The concern expressed around the need for a local residency requirement is one shared by other Australian jurisdictions and is aimed at addressing the threat of “health tourism” (Legal and Constitutional Affairs Committee 2025, 93), evidenced in the operation of the Dignitas assisted dying clinic in Switzerland and the fertility space both globally and within Australia.^6^ The Committee, however, recommended that there be exemptions to both the residency and the citizenship requirements, reflecting a more liberal approach to accessing VAD. For example, the LCAC Report recommends accommodating persons who live outside the Northern Territory but still close to the border and for those individuals who can demonstrate family, cultural or support networks in the Northern Territory (Legal and Constitutional Affairs Committee 2025, 93–94).

The Australian citizenship/residency and state/territory residency provisions differ throughout Australia and has proven to be one of the most fertile areas for legal challenge in relation to VAD (Ricciardo 2024; Del Villar, Jeanneret, and White 2025). In Victoria, Western Australia, and South Australia, a person must be an Australian citizen or permanent resident and must ordinarily reside in the state where they are requesting VAD for at least twelve months at the time of making the first request (*Voluntary Assisted Dying Act 2017* (Vic) s 9(1)(b); *Voluntary Assisted Dying Act 2019* (WA) s 16(1)(b); *Voluntary Assisted Dying Act 2021* (SA) s 26(1)(b)). While Tasmania, Queensland, and New South Wales have retained the same state residency requirements, the rule concerning Australian citizenship or permanent residency is more flexible as persons who have lived in Australia for at least three continuous years before making a first request are eligible to request VAD (*Voluntary Assisted Dying Act 2021* (Qld) s 10(1)(e)(iii); *End of Life Choices* (*Voluntary Assisted Dying) Act 2021* (Tas) s 11(1)(a)(iii); *Voluntary Assisted Dying Act 2022* (NSW) s 16(1)(b)(iii)). Additionally, in New South Wales a state residency exemption may be granted (*Voluntary Assisted Dying Act 2022* (NSW) s 17), and in Queensland, an Australian residency and Queensland residency exemption may be granted in certain circumstances (*Voluntary Assisted Dying Act 2021* (Qld) s 12). In the Australian Capital Territory, no Australian citizenship/residency requirement is imposed, and an exemption may be granted to the twelve-month Territory residency requirement (*Voluntary Assisted Dying Act 2024* (ACT) ss 11(1)(f), 154). The LCAC’s recommendations therefore sit comfortably in the more liberal approaches to citizenship and residency requirements in other VAD frameworks, although the Australian Capital Territory is clearly, on this evidence, the most progressive in affording access to VAD.

## Conclusion

Voluntary assisted dying legislation is expected to be introduced into the NT Legislative Assembly mid-2026 (Chaseling 2025). This brief overview of the recommendations on key eligibility requirements indicates a clear preference for greater access to VAD, reflected in the exclusion of a life expectancy requirement in the definition of the underlying medical condition, and significant exemptions in relation to citizenship and residency requirements. Should these recommendations be ultimately implemented by the Parliament, the Northern Territory would be second only to the Australian Capital Territory in facilitating access to VAD. Given the decades long ban on the Territories introducing this sort of legislation, it seems both fitting and ironic that attempts to control law-making have ultimately resulted in a more progressive outcome.

## Data Availability

Not applicable.

## Footnotes

1 In this paper, reference to “assisted dying” means the administration of a lethal substance to a person and includes both self-administration and practitioner administration. The *Euthanasia Laws Act 1998* (Cth) amended the *Northern Territory (Self-Government) Act 1970* and the *Australian Capital Territory (Self-Government) Act 1988* prohibiting them from “making laws which permit or have the effect of permitting (whether subject to conditions or not) the form of intentional killing of another called euthanasia (which includes mercy killing) or the assisting of a person to terminate his or her life” (schs 1 cl 1, 2 cl 1).

2 This *Recent Developments* article focuses on selected parts of the Legal and Constitutional Affairs Committee’s Final Report. In deciding to limit our discussion to key eligibility criteria, we are not implying that other aspects of the Committee’s report are not important—they are. We simply could not engage in a broader analysis due to word constraints.

3 https://parlinfo.aph.gov.au/parlInfo/download/chamber/hansardr/25920/toc_pdf/House%20of%20Representatives_2022_08_01_Official.pdf;fileType=application%2Fpdf).

4 On this point, the LCAC were supportive of the broad of suffering adopted in Tasmania and the ACT: see *End of Life Choices (Voluntary Assisted Dying) Act* 2021 (Tas) ss 7(g), 14; *Voluntary Assisted Dying Act 2024* (ACT) s 11(1)(c), (4). Cf *Voluntary Assisted Dying Act 2021* (SA) s 26(d)(iv); Voluntary *Assisted Dying Act 2021* (Qld) s 10(1)(a)(iii), (2).

5 In Tasmania, a person may be exempted from the life expectancy requirement stipulated under the Act if it is determined that the “prognosis of the person’s relevant medical condition is such that the paragraph should not apply to them”: *End of Life Choices (Voluntary Assisted Dying) Act 2021* (Tas) s 6(3), (4).

6 See Dignitas 2024. See also Deonandan 2015; *Ex Parte CH* [2023] WASC 487; McGovern 2025.
